# EGFR exon 20 insertion mutations in advanced non-small-cell lung cancer: current status and perspectives

**DOI:** 10.1186/s40364-022-00372-6

**Published:** 2022-04-13

**Authors:** Jiabao Hou, Hongle Li, Shuxiang Ma, Zhen He, Sen Yang, Lidan Hao, Hanqiong Zhou, Zhe Zhang, Jing Han, Li Wang, Qiming Wang

**Affiliations:** 1 Department of Internal Medicine, The Affiliated Cancer Hospital of Zhengzhou University & Henan Cancer Hospital, 127 Dongming Road, Zhengzhou, 450008 China; 2 Department of Molecular Pathology, The Affiliated Cancer Hospital of Zhengzhou University & Henan Cancer Hospital, Zhengzhou, China; 3 Henan Academy of Medical Sciences, 47 Weiwu Road, Zhengzhou, 450008 China

**Keywords:** Lung cancer, EGFR exon 20 insertion mutations, Tyrosine kinase inhibitor, Immune checkpoint inhibitor

## Abstract

Platinum-based chemotherapy was previously the first-choice treatment for lung cancer. The discovery of epidermal growth factor receptor (EGFR) gene mutations and the development of EGFR tyrosine kinase inhibitors (TKIs) marked the beginning of the targeted therapy era for non-small-cell lung cancer (NSCLC). Thirty percent of NSCLC patients carry EGFR gene mutations. For these advanced NSCLC patients, EGFR-TKIs are currently preferred for their superior activity and survival benefits over platinum-based chemotherapy. However, therapeutic efficacy is quite different in patients with EGFR exon 20 insertion (ex20ins) mutations versus common mutations. Patients with ex20ins mutations are insensitive to EGFR-TKIs and have poor prognosis. Some drugs targeting EGFR ex20ins mutations have been approved. Here, we systematically reviewed the recent clinical research of and treatments used for EGFR ex20ins mutations, summarized the latest data on emerging therapies, and discussed future prospects and treatments.

## Introduction

Lung cancer is the leading cause of death in cancer patients worldwide, and non-small-cell lung cancer (NSCLC) accounts for the vast majority (85%) of lung cancer cases, including the histological subtypes squamous cell carcinoma, large cell carcinoma, and adenocarcinoma (most common) [1–4]. Due to the emergence and repurposing of targeted drugs, the treatment effect in lung adenocarcinoma has improved, and the survival period of adenocarcinoma patients has been prolonged. However, unlike patients with lung adenocarcinoma, lung squamous cell carcinoma patients do not benefit from targeted therapy.

Driver gene mutations are present in two-thirds of patients with advanced NSCLC. Targeted therapy is currently available for most patients with driver gene mutations.

Epidermal growth factor receptor (EGFR) mutations are the most frequent type of mutation in NSCLC patients, with approximately 30% of NSCLC patients carrying EGFR mutations [5]. Among NSCLC patients with EGFR mutations, approximately 4–10% of patients have EGFR exon 20 insertion (ex20ins) mutations, with 46% of patients having EGFR exon 19 deletion (ex19del) mutations and 38% of patients having the EGFR L858R point mutation [6]. The demographics of patients with EGFR ex20ins mutations are similar to those of patients with classical EGFR mutations; ex20ins mutations are more common in women, nonsmokers, patients with adenocarcinoma and Asians [7–11].

An observational longitudinal cohort study in Hispanic patients reported that 36.4% of patients had concomitant common EGFR mutations (ex19del/L858R), 8% had G719X/L861Q/S768I mutations and 26% had EGFR amplification. Five patients had additional mutations in PI3K, KRAS and MEK1. Researchers have found that patients with concomitant common EGFR mutations have a better prognosis.

Currently, none of the internationally approved EGFR tyrosine kinase inhibitors (TKIs) have demonstrated adequate antitumor activity as frontline therapy for the treatment of EGFR ex20ins NSCLC. A real-world study showed that NSCLC patients with EGFR ex20ins mutations had lower overall survival (OS) and progression-free survival (PFS) than those with common EGFR mutations (EGFR ex19del and EGFR L858R).

EGFR ex20ins mutations comprise a unique set of EGFR activating mutations. The most common EGFR ex20ins mutations are post C-helix insertions of one to four amino acids, which in aggregate account for 80–90% of all ex20ins mutations. These mutations cause inward movement of the ATP-binding pocket, limiting its binding to traditional TKIs because the insertion site is located at the rear end. The A763_Y764insFQEA isoform with the insertion point located in front of exon 20 is sensitive to erlotinib.

In addition, the results of in vitro experiments have shown that the sensitivity of A763_Y764insFQEA to first- and second-generation TKIs is significantly higher than that of Y764_V765insHH, M766_A767insAI, V769_D770insASV, D770_N771insNPG, D770_N771insNPG, D770_N771ins_SSVD, and H773.

For NSCLC patients carrying rare mutations, timely detection of the type of driver mutation is crucial for subsequent targeted therapy. However, the heterogeneity of EGFR ex20ins mutations is high, and conventional PCR detection methods have a miss rate of more than 50%, so next-generation gene sequencing (NGS) is important for the detection of these mutations. The latest version of the National Comprehensive Cancer Network (NCCN) NSCLC guidelines also emphasizes the importance of NGS testing. Significant progress has been made in recent years in the development of effective targeted therapies for NSCLC patients with EGFR ex20ins mutations. Therefore, we describe the historical challenges and recent advances in this field.

## Chemotherapy

Chemotherapy is still the main treatment option for Chinese patients with advanced EGFR ex20ins NSCLC, but the effect is not satisfactory (Table 1).

**Table 1:** Retrospective studies of patients with EGFR ex20ins NSCLC treated with chemotherapy

Treatment (reference)	N	Line	ORR (%)	mPFS (months)	mOS (months)
Platinum-based chemotherapy [12]	105	1	19.2	6.4	NA
Platinum-based chemotherapy [13]	21	1	19.0	6.5	NA
Platinum-based chemotherapy [14]	49	1	NA	7.6	19.9
Pemetrexed-based chemotherapy [15]	77	1	41.6	5.5	25

A retrospective study included a total of 105 patients with EGFR ex20ins NSCLC who received platinum-based chemotherapy as first-line treatment. The overall response rate (ORR) was 19.2%, and the disease control rate (DCR) at 6 months was 41.3%. The median PFS was 6.4 months [12]. Shi Jinpeng et al. found that 21 patients with mutations who received first-line platinum-based chemotherapy had an ORR of 19.0% and a median PFS (mPFS) of 6.5 months [13]. In a retrospective study that included 49 patients with mutations who received first-line platinum-based chemotherapy, the mPFS was 7.6 months, and the median OS (mOS) was 19.9 months [14].

In a retrospective study, a total of 77 patients received pemetrexed-based first-line chemotherapy, with an mPFS of 5.5 months and an mOS of 25 months. Researchers also found that pemetrexed-based chemotherapy can control disease better than non-pemetrexed-based chemotherapy [15].

## EGFR-TKIs and combination therapy

EGFR is a tyrosine kinase receptor that is widely distributed in cell membranes in human tissues and can regulate various signaling processes, such as cell proliferation, metastasis and apoptosis. Mutations in the EGFR gene can lead to continuous activation of its downstream signaling pathways, promoting tumor cell proliferation and inhibiting tumor cell apoptosis [16, 17]. For NSCLC patients carrying EGFR gene mutations, the main clinical treatment is currently TKIs, such as the first-generation TKIs gefitinib, erlotinib and icotinib; the second-generation TKIs afatinib and dacomitinib; and the third-generation TKI osimertinib [18–22].

These drugs covalently bind to the kinase structural domain of EGFR, thereby inhibiting the activation of aberrant signaling. EGFR mutations are mainly found in exons 18, 19, 20 and 21. EGFR ex19del mutations and EGFR exon 21 L858R point mutations are classical mutations that are sensitive to EGFR-TKI treatment. However, ex20ins mutations are less sensitive to EGFR-TKIs [23, 24] (Table 2).

**Table 2:** Retrospective studies of patients with EGFR ex20ins NSCLC treated with EGFR-TKIs and combination therapy

Treatment (reference)	N	Line	ORR (%)	mPFS (months)	mOS (months)
Erlotinib/gefitinib [25]	25	≥2	8	2	9.5
Afatinib [26]	23	1	8.7	2.7	9.2
Osimertinib 80 mg [27]	6	≥2	66.7	6.2	NA
Osimertinib 80 mg [28]	15	≥2	0	3.5	56.3
Osimertinib 160 mg [29]	20	≥2	25	9.7	NA
Luminespib [30]	29	≥2	17	2.9	12.8
Afatinib combined with cetuximab [31]	4	≥2	75	5.4	NA

EGFR ex20ins mutations have different subtypes; 80–90% of mutations have an insertion site at the back of exon 20, and they have primary resistance to first- and second-generation TKIs. However, a small number of mutations feature an insertion site at the front end; for example, the A763_Y764insFQEA mutation is sensitive to erlotinib [32–34].

### Erlotinib, gefitinib, and afatinib

A large multicenter study of rare EGFR mutations in France showed that among 25 patients with EGFR ex20ins mutations who received erlotinib/gefitinib, the mOS was 9.5 months, the mPFS was 2 months, and the ORR was 8% [25].

A combined post hoc analysis of LUX-Lung 2, LUX-Lung 3, and LUX-Lung 6 data showed that the mOS and mPFS in 23 patients with EGFR ex20ins mutations who received afatinib were 9.2 and 2.7 months, respectively [26].

### Osimertinib

Osimertinib is a third-generation EGFR-TKI that was developed for sensitive EGFR mutations and EGFR T790M resistance mutations. Preclinical studies have reported that osimertinib is effective in EGFR ex20ins mutant cell lines and tumor xenografts with a wide therapeutic window [35–38].

In one study that included 6 patients with EGFR ex20ins mutations, the patients received 80 mg of osimertinib daily. The investigators evaluated the antitumor activity and safety of osimertinib monotherapy. They found that four patients achieved partial response (PR), and the remaining patients achieved stable disease (SD). The mPFS was 6.2 months (95% confidence interval 5.0–12.9 months; range 4.9–14.6 months) [27].

A multicenter phase II clinical trial from the Korean Cancer Research Group (LU17–19) aimed to evaluate the efficacy of osimertinib in NSCLC patients with EGFR ex20ins mutations after standard chemotherapy failed. The study enrolled 15 patients with EGFR ex20ins mutations who were treated with osimertinib; 3 patients (20%) received osimertinib as second-line treatment and 12 patients (80%) received osimertinib as third-line or beyond treatment. Among the patients, 46.7% (*n* = 7) achieved SD. Three patients were still receiving osimertinib at the time of data cutoff. The patients’ mPFS was 3.5 months, and the DCR at 6 months was 31.1% [28].

Piotrowska Zofia et al. conducted a single-arm, phase II study of osimertinib 160 mg in NSCLC patients with EGFR ex20ins mutations. Among the 20 eligible patients, the best response was PR in 4 patients and complete response (CR) in one patient, for a confirmed ORR of 25%; 12 (60%) patients achieved SD. The mPFS was 9.7 months, and the median duration of response (DOR) was 5.7 months. Osimertinib (160 mg daily) was well tolerated and showed clinical activity in EGFR ex20ins mutant NSCLC [29]. This result suggests that high-dose osimertinib may be used as a well-tolerated treatment option for patients. Of course, the result needs to be verified in large-scale clinical trials. Increasing the dose of targeted drugs could provide more benefit for the treatment of NSCLC patients with EGFR ex20ins mutations.

### Luminespib

A preclinical study found that EGFR exon 20 mutations were dependent on Hsp90 and could be inhibited by the Hsp90 inhibitor luminespib [39]. This phase II trial enrolled 29 patients with EGFR ex20ins NSCLC. The results showed that the mPFS and OS were 2.9 months and 12.8 months, respectively, and the most common treatment-related adverse events (TRAEs) were diarrhea (83%), visual changes (76%) and fatigue (45%) [30].

### Cetuximab and afatinib

A study using cytological in vitro and in vivo experiments in tumor-bearing mice found that a combination of the EGFR monoclonal antibody (mAb) cetuximab and afatinib inhibited the growth of cells or transplanted tumors carrying EGFR exon 20 partial insertion mutations and promoted apoptosis of cancer cells [40]. A study in Finland used afatinib combined with cetuximab in four patients, and three of the four patients achieved PR. The mPFS was 5.4 months. Preliminary clinical studies have shown that afatinib combined with cetuximab is more effective than monotherapy [31].

## Mechanisms of the poor efficacy of conventional EGFR-TKIs

Previous studies have found that the reason for the poor efficacy of conventional EGFR-TKIs is alterations in the drug-binding pocket of exon 20. Jacqulyne P. Robichaux et al. visualized these changes through 3D modeling. Their modeling showed that EGFR ex20ins mutations result in a shift of the phosphate-binding loop (P-loop) into the drug-binding pocket and increased affinity for ATP. The steric hindrance caused by these shifts reduces the binding of first-generation inhibitors, thereby making these mutants resistant to noncovalent inhibitors.

In addition, a large terminal 1-methylindole group is attached to the pyrimidine core of osimertinib, which reduces the ability of osimertinib to efficiently reach the Cys797 residue in the EGFR ex20ins mutation. In vitro data and computer simulations suggest that small, flexible quinazoline derivatives may be able to target EGFR ex20ins mutants.

## Immunotherapy

Immune checkpoint inhibitors (ICIs), such as PD-1/PD-L1 mAbs, have brought unprecedented and lasting clinical benefits for NSCLC patients, but the response rate for patients with driver gene mutations is still relatively low [4].

One meta-analysis included studies such as CheckMate 057, KEYNOTE-010, and POPLAR. The analysis compared nivolumab, pembrolizumab, and atezolizumab with docetaxel, and the results showed that 186 patients with EGFR mutations did not seem to benefit from immunotherapy, which is in sharp contrast with the 1362 patients with wild-type EGFR who did benefit [41].

For patients with EGFR ex20ins mutations, the underlying mechanism of the PD-1/PD-L1 inhibitor response remains unclear. In one study, 24 patients with EGFR mutations were treated with nivolumab. Analysis of the results showed that rare mutations were a predictor of better efficacy [42].

ICIs are not effective in NSCLC with EGFR ex20ins mutations, especially as a first-line treatment. A retrospective study included 30 NSCLC patients with EGFR ex20ins mutations, of whom 15 had been treated with ICIs. Compared with patients who did not receive immunotherapy, patients who received ICIs had a shorter mOS [43].

A real-world study in the United States showed that regardless of treatment line, the treatment endpoints (ORR, OS, and PFS) of patients who received single-agent immunotherapy were not as good as those of patients who received platinum-containing chemotherapy or immunotherapy combined with platinum-containing chemotherapy [44] (Table 3).

**Table 3:** Studies of immunotherapy in patients with EGFR ex20ins NSCLC

Treatment (reference)	N	Line	ORR (%)	mPFS (months)	mOS (months)
ICI (monotherapy or combination therapy) [43]	15	≥1	6.7	2.0	5.3
ICI monotherapy [44]	11	1	9.1	11.0	3.1
ICI + platinum agent [44]	16	1	18.8	11.3	4.5
ICI monotherapy [44]	32	≥2	3.1	8.1	2.3
ICI + platinum agent [44]	20	≥2	5	7.1	2.2

In patients with EGFR mutations, higher PD-L1 expression seems to be related to longer PFS. However, the immune environment of EGFR mutant tumors is very complex, and the use of factors such as PD-L1 to predict immune efficacy is far from sufficient. A variety of immune parameters have been found to be related to the response of EGFR mutant tumors to immunotherapy, including PD-L1 expression, tumor mutation burden (TMB), the human leukocyte antigen (HLA)-T-cell receptor (TCR) axis, tumor metabolic factors, tumor-infiltrating lymphocytes (TILs), immune cell infiltration, and soluble molecules [45].

A retrospective study in China included 35 NSCLC patients with EGFR ex20ins mutations and reported their immune microenvironment features. A total of 48.6% of the patients (17/35) had positive PD-L1 expression, and PD-L1 was identified as an independent predictive factor [46]. An observational longitudinal cohort study enrolled 88 Hispanic NSCLC patients with EGFR ex20ins mutations. The results of the study showed that the average PD-L1 expression in this population appeared to be higher than that of patients with common EGFR mutations, and 81.7% of the patients had positive PD-L1 expression (1–50%) [47]. A recent study described the treatment outcomes and immunophenotypic characteristics of 6290 NSCLC patients. The proportion of tumors with PD-L1 expression ≥1% (22% vs. 60%; *p* < 0.001) was lower in patients with EGFR ex20ins mutations than in patients with other forms of NSCLC [48].

In different studies of EGFR ex20ins mutations, the expression of PD-L1 varies greatly, which may be related to the antibody and detection technology employed. However, these discrepancies seem to imply that the PD-L1 expression level alone cannot predict the response of patients with EGFR ex20ins mutations to immunotherapy.

Previous studies have shown that a higher TMB can enhance the immunogenicity of tumors by increasing the number of new antigens, which is related to better immunotherapy effects [49–52]. Compared with wild-type patients, patients with genetic driver mutations such as EGFR mutations have significantly lower TMB [53]. Analysis of The Cancer Genome Atlas (TCGA) TMB data showed that the mutation load was relatively lower in patients with EGFR mutation than in patients with wild-type EGFR (median TMB: 56 vs. 181, *p* < 0.001). The median TMB was lower in EGFR ex20ins patients than in other NSCLC patients (3.5 vs. 5.9, 10 p < 0.001) [48]. Lower TMB levels also seem to predict poor immunotherapy response.

TILs are important cells that can infiltrate tumor nests and stroma to exert a killing effect. A higher density of CD8+ TILs is associated with better clinical outcomes [54, 55]. However, in patients with EGFR ex20ins mutations, neither CD4+ TILs nor CD8+ TILs show predictive value (*p* = 0.503 and *p* = 0.095, respectively) [46].

However, clinical trials of immunotherapy in patients with EGFR ex20ins mutations are lacking. Determining whether PD-1/PD-L1 inhibitors can bring greater benefits to patients with EGFR ex20ins mutations than chemotherapy requires further clinical studies.

## Emerging therapies and ongoing clinical trials

**Fig. 1:**
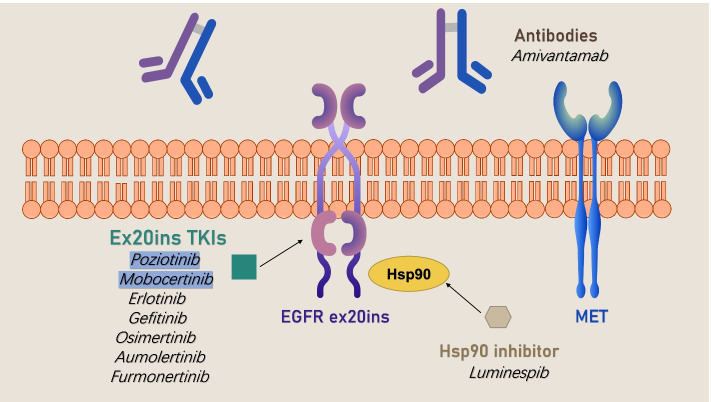
Emerging therapies in EGFR ex20ins NSCLC. Conventional EGFR-TKIs have shown limited efficacy, such as erlotinib, gefitinib and afatinib. Combination therapy has become a trend in some clinical trials. Currently known and relatively mature drugs include mobocertinib, amivantamab, poziotinib and furmonertinib

For patients with EGFR ex20ins mutations, a variety of new drugs are currently in development. Combination therapy has become a trend in drug development, and some clinical trials are ongoing. Currently known and relatively mature drugs include mobocertinib (TAK-788), amivantamab (JNJ372), poziotinib and furmonertinib (Tables 4 and 5 and Fig. 1).

**Table 4:** Latest results of studies of emerging therapies

Treatment (reference)	N	Line	ORR (%)	mPFS (months)	mOS (months)
Poziotinib [56]	115	≥2	14.8	4.2	NA
Mobocertinib [57]	114	≥2	28	7.2	24
Amivantamab [58]	81	≥2	40	8.3	22.8
CLN-081 [59]	42	≥2	31	NA	NA
DZD9008 [60]	56	≥2	37.5	NA	NA
Furmonertinib [61]	10	≥2	60	NA	NA

**Table 5:** Summary of ongoing clinical trials for non-small cell lung cancer patients with EGFR exon 20 insertion mutations

Phase	Title	Treatment	Primary Outcome	Enrollment^a^	Status	NCT Number
I	Study of FURMONERTINIB in Patients With NSCLC Having Exon 20 Insertion Mutation	Furmonertinib	ORR	30	Recruiting	NCT04858958
I/II	A Study of BDTX-189, an Orally Available Allosteric ErbB Inhibitor, in Patients With Advanced Solid Tumors.	BDTX-189	Phase II:ORR	200	Recruiting	NCT04209465
I/II	A Phase 1/2a Trial of CLN-081 in Patients With Non-Small Cell Lung Cancer	CLN-081	Safety and Tolerability	80	Recruiting	NCT04036682
I/II	Assessing an Oral EGFR Inhibitor, DZD9008 in Patients Who Have Advanced Non-small Cell Lung Cancer With EGFR or HER2 Mutation (WU-KONG1)	DZD9008	Part B:ORR	220	Recruiting	NCT03974022
I/II	A Study of FWD1509 in Adults With Non-Small Cell Lung Cancer	FWD1509	Safety and Tolerability	130	Recruiting	NCT05068024
I/II	Study of Poziotinib in Japanese Patients With NSCLC	Poziotinib	Phase II:ORR	76	Recruiting	NCT04402008
I/II	A Study of TAK-788 in Adults With Non-Small Cell Lung Cancer	TAK-788	ORR	395	Recruiting	NCT02716116
II	Afatinib and Cetuximab in Epidermal Growth Factor Receptor (EGFR) Exon 20 Insertion Positive Non-small-cell Lung Cancer	Afatinib plus cetuximab	DCR after 18 weeks	37	Recruiting	NCT03727724
II	Almonertinib as Upfront Treatment for Uncommon EGFR Mutation Harboring Non-Small-Cell Lung Cancer Patients: A Multicenter, Open-Label, Phase II Trial	Almonertinib	ORR	53	Not yet recruiting	NCT04553887
II	Study of Amivantamab, a Human Bispecific EGFR and cMet Antibody, in Participants With Advanced Non-Small Cell Lung Cancer	Amivantamab	ORR and DOR	780	Recruiting	NCT02609776
II	First in Human Study of BAY2927088 in Participants Who Have Advanced Non-small Cell Lung Cancer (NSCLC) With Mutations in the Genes of Epidermal Growth Factor Receptor (EGFR) and/or Human Epidermal Growth Factor Receptor 2 (HER2)	BAY2927088	Safety and Tolerability	250	Not yet recruiting	NCT05099172
II	Dacomitinib in Lung Cancer With Uncommon EGFR Mutations	Dacomitinib	ORR	30	Recruiting	NCT04504071
II	Efficacy and Safety of JMT101 Combined With Afatinib (or Osimertinib) in Patients With Non-Small Cell Lung Cancer	JMT101 Combined With Afatinib (or Osimertinib)	Safety	48	Recruiting	NCT04448379
II	A Study of Lazertinib as Monotherapy or in Combination With Amivantamab in Participants With Advanced Non-small Cell Lung Cancer	Lazertinib and Amivantamab	Safety and Tolerability	520	Recruiting	NCT04077463
II	A Study Osimertinib in Patients With Stage 4 Non-small Cell Lung Cancer With Uncommon EGFR Mutations	Osimertinib	ORR	37	Recruiting	NCT03434418
II	Osimertinib Combined With Bevacizumab in the Treatment Epidermal Growth Factor Receptor (EGFR) Exon 20 Insertions Metastatic Non-Small Cell Lung Cancer	Osimertinib combined with Bevacizumab	PFS	20	Recruiting	NCT04974879
II	Poziotinib in EGFR Exon 20 Mutant Advanced NSCLC	Poziotinib	ORR	80	Recruiting	NCT03066206
II	Phase 2 Study of Poziotinib in Patients With NSCLC Having EGFR or HER2 Exon 20 Insertion Mutation	Poziotinib	ORR	603	Recruiting	NCT03318939
II	Poziotinib and Ramucirumab for the Treatment of EGFR Exon 20 Mutant Stage IV Non-small Cell Lung Cancer	Poziotinib and Ramucirumab	PFS	36	Not yet recruiting	NCT05045404
II	Study of Tarloxotinib in Pts With NSCLC (EGFR Exon 20 Insertion, HER2-activating Mutations) & Other Solid Tumors With NRG1/ERBB Gene Fusions	Tarloxotinib	ORR	60	Terminated	NCT03805841
II/III	A Study to Evaluate Efficacy and Safety of Multiple Targeted Therapies as Treatments for Participants With Non-Small Cell Lung Cancer (NSCLC)	Atezolizumab, Bevacizumab, Carboplatin, and Pemetrexed	ORR	700	Recruiting	NCT03178552
III	TAK-788 as First-line Treatment Versus Platinum-Based Chemotherapy for Non-Small Cell Lung Cancer (NSCLC) With EGFR Exon 20 Insertion Mutations	TAK-788 Versus Platinum-Based Chemotherapy	PFS	318	Recruiting	NCT04129502
III	A Study to Evaluate the Efficacy and Safety of Toripalimab or Placebo Combined With Chemotherapy in Treatment-naive Advanced NSCLC	Toripalimab combined with standard chemotherapy	PFS	450	Recruiting	NCT03856411
III	A Study of Combination Amivantamab and Carboplatin-Pemetrexed Therapy, Compared With Carboplatin-Pemetrexed, in Participants With Advanced or Metastatic Non-Small Cell Lung Cancer Characterized by Epidermal Growth Factor Receptor (EGFR) Exon 20 Insertions	Amivantamab + Chemotherapy Versus Chemotherapy Alone	PFS	300	Recruiting	NCT04538664

*Abbreviations*: *NSCLC* Non-small cell lung cancer, *EGFR* Epidermal growth factor receptor, *ex* Exon, *ins* Insertion, *ORR* Objective response rate, *DCR* Disease control rate, *DOR* Duration of response, *PFS* Progress free survival

^a^Numbers for total study was given

### Poziotinib

Poziotinib is an orally available quinazoline-based EGFR inhibitor. Jacqulyne P. Robichaux et al. used in vitro and in vivo experiments to simulate the structural changes caused by exon 20 mutations, and 3D modeling showed a decreased size of the drug-binding pocket. Poziotinib has advantages in size and structure compared to other EGFR-TKIs. Its smaller terminal and substituent linkers make it more flexible. Moreover, poziotinib can overcome the effects of steric hindrance and bind tightly to the drug-binding pocket. In xenograft models derived from patients with EGFR or HER2 exon 20 mutant NSCLC and genetically engineered NSCLC mouse models in vitro, poziotinib showed stronger activity than approved EGFR-TKIs [62]. ZENITH20, a multicenter, multicohort phase II clinical trial, was designed to evaluate the efficacy and safety of poziotinib in NSCLC patients with EGFR or HER2 ex20ins mutations. The cohort 1 study included 115 NSCLC patients with EGFR ex20ins mutations who had previously received treatment with poziotinib. The ORR and DCR were 14.8 and 69%, respectively. The mPFS and median DOR were 4.2 and 7.4 months, respectively. Although the ZENITH20 cohort 1 study did not meet the primary endpoint of ORR, poziotinib did shrink tumors in most (65%) patients. The cohort 3 study included 79 patients with EGFR ex20ins mutations who were treated with first-line poziotinib. The ORR was 27.8%, the DCR was 86.1%, the mPFS was 7.2 months, and the median DOR was 9.2 months [56].

### Mobocertinib (TAK-788)

Mobocertinib is an oral TKI specifically designed to selectively target EGFR ex20ins mutations. Determining which of the residues to target was difficult due to the absence of amino acid substitutions at the binding site. However, the researchers found in a docking model that an unoccupied pocket remains after binding of the EGFR ex20insNPG mutant to osimertinib, which can be accessed by substitution at the pyrimidine ring. Therefore, the isopropyl ester in mobocertinib was specifically designed to interact with the gatekeeper residues within this pocket. Furthermore, mobocertinib binds irreversibly to the Cys797 residue in EGFR, forming a covalent interaction. This bond has a higher affinity. In October 2020, mobocertinib was awarded “Breakthrough Therapy Certification” in China. In July 2021, the results of a phase 1/2 trial of mobocertinib were accepted by the Center for Drug Evaluation (CDE) of the National Medical Products Administration of China and are under review. On September 15, 2021, the US Food and Drug Administration (FDA) approved the application of mobocertinib for adult patients with locally advanced or metastatic NSCLC with EGFR ex20ins mutations. The approval was based on data from patients who had previously received platinum-based chemotherapy in a phase 1/2 trial of mobocertinib. The trial enrolled 114 patients with EGFR ex20ins mutation-positive NSCLC who had previously received platinum-based chemotherapy. These patients received treatment at a dose of 160 mg. The results of the phase 1/2 trial were announced at the 2021 American Society of Clinical Oncology (ASCO) annual meeting. The ORR confirmed by the Independent Review Committee (IRC) was 28% (the ORR determined by the investigators was 35%). The median DOR confirmed by the IRC was 17.5 months, the mOS was 24 months, the mPFS confirmed by the IRC was 7.3 months, and the DCR reached 78%. The safety was similar to that of other EGFR-TKIs. A total of 22–25% of the patients developed adverse events (AEs) that required dose adjustment. A total of 10–17% of the patients stopped treatment due to AEs [57].

### Amivantamab (JNJ-372)

Amivantamab is a fully human EGFR/MET bispecific mAb. It has two binding arms (a monovalent arm that binds to EGFR and another monovalent arm that binds to MET). Amivantamab is capable of blocking ligand-induced phosphorylation of EGFR and cMet, thus blocking the activation of the signaling pathway, transmitting signals downstream, and inhibiting the proliferation of tumor cells expressing relevant molecules.

Amivantamab also has immune cell-targeting activity and is designed to interfere with two different signaling pathways. By binding to the extracellular domain of each receptor, amivantamab can inhibit ligand binding, promote the endocytosis and degradation of receptor-antibody complexes, and induce Fc-dependent phagocytosis of macrophages and antibody-dependent natural killer cell cytotoxicity [63–65].

CHRYSALIS was the first phase I clinical trial conducted in humans to evaluate the efficacy, safety and pharmacokinetics of amivantamab in patients with advanced NSCLC. Patients with EGFR ex20ins mutations who had previously received platinum-containing chemotherapy were recruited for the study. The results showed that the mOS was 22.8 months, the mPFS was 8.3 months, and the ORR was 40% in the amivantamab group [58, 66]. Based on these results, the FDA approved amivantamab for the treatment of adult patients with EGFR ex20ins mutations who experience progression after failure of platinum-containing chemotherapy.

### CLN-081

CLN-081 is a new oral EGFR-TKI that has a wide range of activities against clinically relevant EGFR mutations (including ex20ins mutations). In vitro, the activity of wild-type EGFR is weaker than that of EGFR ex20ins mutants, suggesting that CLN-081 has a more favorable clinical treatment window [67, 68]. In a trial evaluating CLN-081, patients with EGFR ex20ins who had previously received platinum-based therapy were enrolled and received CLN-081 twice a day (BID) for 21 days as a cycle. The doses included 30 mg, 45 mg, 65 mg, 100 mg, and 150 mg. Ninety-eight percent (41/42) of patients achieved a best response of SD or PR. Seventy-six percent of patients had tumor shrinkage at the first assessment. Responses were also seen in the group of patients who had previously received ICIs or EGFR-TKIs.

Moreover, the symptoms reported by patients, such as dyspnea, shortness of breath, and coughing, could be rapidly improved [59].

### DZD9008

DZD9008 is a small molecule compound designed for EGFR/HER2 ex20ins mutant cancer. James Chih-Hsin Yang et al. explored the efficacy and safety of DZD9008 in NSCLC patients with EGFR ex20ins mutation, and the results showed that DZD9008 has good safety and antitumor efficacy in EGFR ex20ins NSCLC patients. Overall, 56 patients with EGFR ex20ins mutations who underwent efficacy evaluation had more than 16 different insertion mutations. DZD9008 had antitumor activity in patients with different EGFR ex20ins mutations. In all 56 patients with EGFR ex20ins mutations, the ORR reached 37.5% (21/56), and the DCR reached 85.7% (48/56). Among the different dose groups, the 200 mg dose group had the highest ORR (45.5%; 5/11); the 300 mg dose group had an ORR of 41.9% (13/31), and the DCR reached 90.3% (28/31) [60]. The researchers plan to further explore the impact of the two doses (200 mg and 300 mg) on the survival benefits of patients.

### Furmonertinib

Furmonertinib has a more innovative structure than osimertinib. Compared with osimertinib, which contains an anisole, furmonertinib has a more hydrophobic trifluoroethoxypyridine structure. In the binding region, the concave hydrophobic pocket composed of the hydrophobic amino acids L792 and M793 has higher affinity, which improves the activity of the drug itself. The initial results of the FAVOUR study showed that in advanced EGFR ex20ins NSCLC patients treated with first-line furmonertinib, the IRC-confirmed ORR was 60%, the ORR determined by the investigators was 70%, and the DCR was 100%. Preliminary reports of furmonertinib therapy have shown good efficacy and safety. Based on these findings, further clinical research on furmonertinib combined with chemotherapy, drugs targeting other pathways and antiangiogenic drugs should be carried out in the future. In addition, further exploration of the study data is ongoing [61].

## Safety overview

**Fig. 2:**
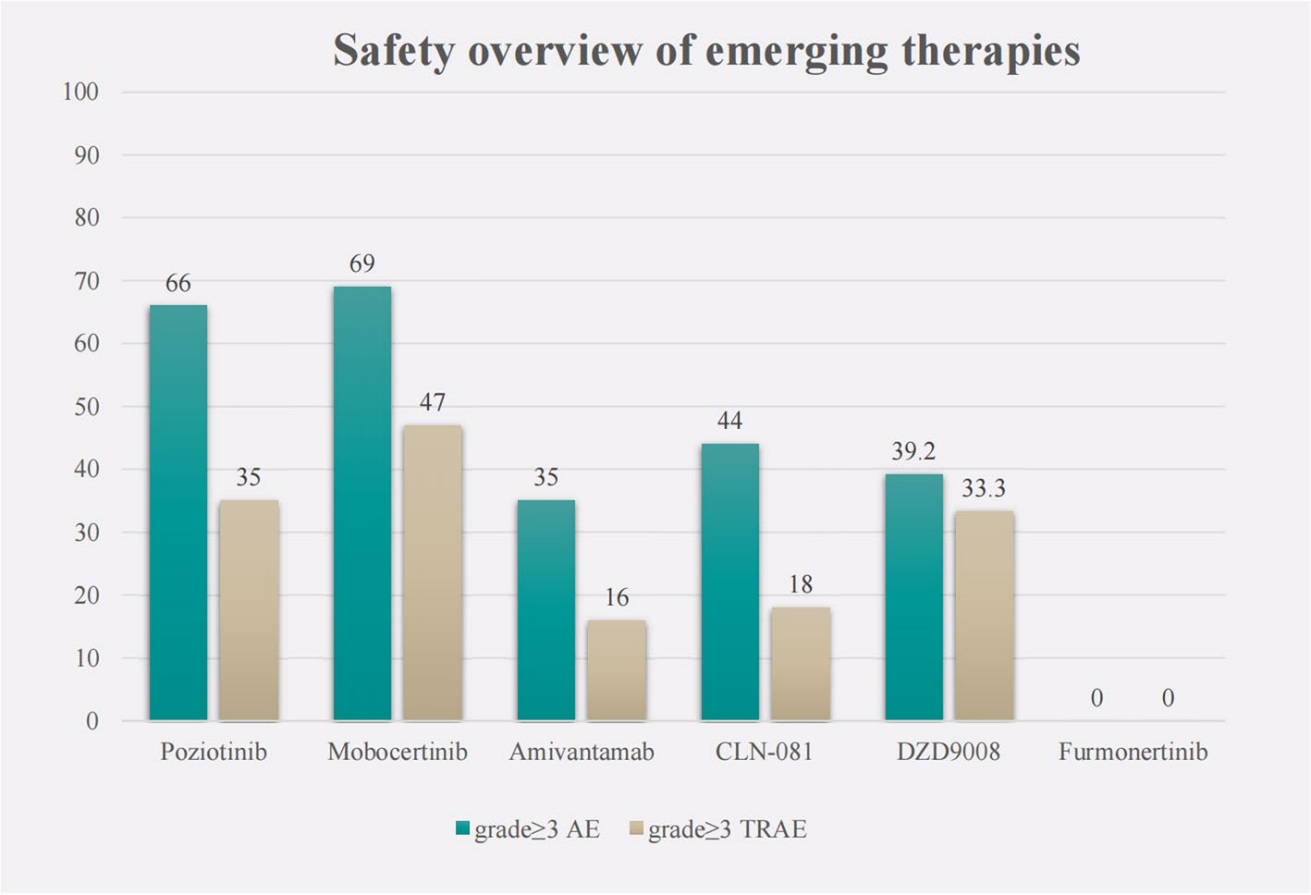
Safety overview of emerging therapies. The AEs (grade ≥ 3) of poziotinib, mobocertinib, amivantamab, CLN-081and DZD9008 were 66, 69, 35, 44 and 39.2%, respectively. The TRAEs (grade ≥ 3) of poziotinib, mobocertinib, amivantamab, CLN-081and DZD9008 are 35%, 47, 16, 18 and 33.3%, respectively. Furmonertinib is relatively safe. AEs or TRAEs (grade ≥ 3) were not observed in the cohort

In terms of safety, a clinical trial of poziotinib showed that the proportions of patients with treatment interruption and dose reduction due to AEs were 89 and 76%, respectively. The incidence of AEs (grade ≥ 3) was 66%, and rash was the most common AE, with an incidence of 50%. The most common grade 3 or 4 TRAEs of mobocertinib were diarrhea (21%), nausea (4%), and stomatitis (4%). The most common any-grade TRAEs were diarrhea (91%), rash (45%), and paronychia (38%). For amivantamab, the AEs related to blockade of the EGFR pathway included rash (86%), paronychia (45%), oral mucositis (21%), and diarrhea (12%); the AEs related to MET pathway blockade included hypoalbuminemia (27%) and peripheral edema (18%). Grade 3 or higher AEs occurred in 35% of patients, the most common being hypokalemia (6%). TRAEs led to dose reductions in 13% of patients, and 4% of patients permanently discontinued treatment. Any-grade TRAEs occurred in 98% of patients treated with CLN-081, and 18% of patients had grade 3 or higher TRAEs, including anemia (9%), increased alanine aminotransferase (4%), increased aspartate aminotransferase (4%), diarrhea (2%), increased amylase (2%), neutropenia (2%), and stomatitis (2%). Discontinuation due to TRAEs was needed in 9% of patients. Dose reductions were needed in 11% of patients. For DZD9008, the most common AEs during treatment were diarrhea (53.9% all grades, 4.9% grade 3+), rash (40.2% all grades, 0 grade 3+), and nausea (34.3% all grades, 0 grade 3+). Furmonertinib is relatively safe. Nine enrolled patients experienced TRAEs, and the most common TRAE was diarrhea. One patient discontinued treatment for 6 days due to diarrhea. No AEs (grade ≥ 3) were observed in the cohort (Fig. 2).

## Other combination therapies and ongoing research

At present, platinum-based chemotherapy is still the most commonly used first-line treatment for patients with EGFR ex20ins NSCLC, and the efficacy of existing TKIs is not satisfactory. Several clinical trials designed to evaluate the safety and efficacy of combination therapy are underway. For example, osimertinib combined with bevacizumab, afatinib plus cetuximab, and amivantamab plus chemotherapy are being tested. The final results of these studies are still awaited (Table 5).

## Summary and prospects

The proportion of patients with EGFR ex20ins mutations is not large. Regardless, given the number of lung cancer patients in China and the world, these patients still represent a substantial group that cannot be ignored. If an effective treatment strategy can be found, it will undoubtedly improve the overall quality of life of lung cancer patients.

In recent years, antibody−drug conjugates (ADCs) have achieved substantial progress in the treatment of solid tumors, especially for lung cancer. ADCs exploit three main components: antigens, antibodies and small molecule toxic drugs. Antigens have high homogeneous expression in tumor cells, and antibodies have high affinity for tumor antigens and reduced immunogenicity through chimerism or humanization. ADCs selectively deliver effective cytotoxic drugs to antigen-expressing tumor cells by exploiting the properties of mAbs. MRG003 is a novel ADC composed of a humanized anti-EGFR mAb conjugated to MMAE via a vclinker. A phase I study of MRG003 demonstrated a manageable safety profile and showed encouraging antitumor activity in patients with advanced pretreated solid tumors, including patients with nasopharyngeal carcinoma and squamous cell carcinoma of the head and neck. If population stratification can be achieved, it will be possible to develop new treatments for patients with EGFR ex20ins mutations in the future. A double-dose osimertinib regimen was well tolerated and demonstrated clinical activity. Although the results need to be verified in large-scale clinical trials, increasing the dose of targeted drugs undoubtedly provides benefits for the treatment of NSCLC patients with EGFR ex20ins mutations. In addition, combination therapy is worthy of basic and clinical exploration in these patients.

Both amivantamab and mobocertinib are FDA-approved for the treatment of adult patients with locally advanced or metastatic NSCLC harboring EGFR ex20ins mutations that has progressed after prior chemotherapy. Although these two drugs are currently approved for the second-line treatment of EGFR ex20ins mutant NSCLC, chemotherapy is still the standard first-line treatment commonly used globally. More treatment strategies, especially first-line treatment strategies, still need to be explored.

The treatment of patients with EGFR ex20ins mutations is still challenging. On the one hand, there are problems related to the detection of EGFR ex20ins mutations. The detection rate is low due to the diversity and complex structures of the insertions. Data from Foundation Medicine showed that 51.4% of mutations were missed by PCR compared to NGS. NGS provides more comprehensive detection of EGFR ex20ins mutations, but there are not yet detection standards or specifications. On the other hand, most of the currently approved indications and completed clinical studies have been in second-line and later settings, and there is a substantial unmet need for first-line treatments for patients. Finally, the mechanisms of targeted drug resistance, for example, the mechanism of resistance to first-line/second-line mobocertinib, are worthy of attention, as are the incidences of AEs and the treatment options after drug resistance. In view of the above problems, further in-depth basic research is needed.

## Data Availability

All data generated or analyzed during this study are included in this published article.

## Abbreviations

EGFR: Epidermal growth factor receptor
EGFR-TKIs: EGFR tyrosine kinase inhibitors
NGS: Next-generation sequencing
NSCLC: Non-small-cell lung cancer
ex: Exon
ins: Insertion
ORR: Objective response rate
DCR: Disease control rate
DOR: Duration of response
PFS: Progression-free survival
